# Referral criteria for chronic kidney disease: implications for disease management and healthcare expenditure—analysis of a population-based sample

**DOI:** 10.1186/s12882-022-02845-0

**Published:** 2022-06-24

**Authors:** Simone Kiel, Gesine Weckmann, Jean-François Chenot, Sylvia Stracke, Jacob Spallek, Aniela Angelow

**Affiliations:** 1 Department of General Practice, Institute for Community Medicine, University Medicine Greifswald, Fleischmannstrasse 6, Greifswald, 17475 Germany; 2 Faculty of Applied Health Sciences, European University of Applied Sciences, Rostock, Germany; 3 Department of Internal Medicine A, Nephrology, University Medicine Greifswald, Greifswald, Germany; 4 KfH Kidney Center Greifswald, Greifswald, Germany; 5 Department of Public Health, Brandenburg University of Technology Cottbus- Senftenberg, Senftenberg, Germany

**Keywords:** Chronic kidney disease, Referral, Guideline recommendations, Nephrology referral, Health care costs

## Abstract

**Background:**

Clinical practice guidelines recommend specialist referral according to different criteria. The aim was to assess recommended and observed referral rate and health care expenditure according to recommendations from:

• Kidney Disease Improving Global Outcomes (KDIGO,2012)

• National Institute for Health and Care Excellence (NICE,2014)

• German Society of Nephrology/German Society of Internal Medicine (DGfN/DGIM,2015)

• German College of General Practitioners and Family Physicians (DEGAM,2019)

• Kidney failure risk equation (NICE,2021)

**Methods:**

Data of the population-based cohort *Study of Health in Pomerania* were matched with claims data. Proportion of subjects meeting referral criteria and corresponding health care expenditures were calculated and projected to the population of Mecklenburg-Vorpommern.

**Results:**

Data from 1927 subjects were analysed. Overall proportion of subjects meeting referral criteria ranged from 4.9% (DEGAM) to 8.3% (DGfN/DGIM). The majority of patients eligible for referral were ≥ 60 years. In subjects older than 60 years, differences were even more pronounced, and rates ranged from 9.7% (DEGAM) to 16.5% (DGfN/DGIM). Estimated population level costs varied between €1,432,440 (DEGAM) and €2,386,186 (DGfN/DGIM). From 190 patients with eGFR < 60 ml/min, 15 had a risk of end stage renal disease > 5% within the next 5 years.

**Conclusions:**

Applying different referral criteria results in different referral rates and costs. Referral rates exceed actually observed consultation rates. Criteria need to be evaluated in terms of available workforce, resources and regarding over- and underutilization of nephrology services.

**Supplementary Information:**

The online version contains supplementary material available at 10.1186/s12882-022-02845-0.

## Introduction

Chronic kidney disease (CKD) has a prevalence of approximately 9% in adults worldwide [1]. Glomerular filtration rate (GFR) is used to grade kidney function in stages G1-G5. Due to age-dependent decline of kidney function [2], the prevalence of CKD stages 3–5 increases with age and reaches up to 45% in the age group 75–84 years [3]. GFR and proteinuria can be used to grade the severity of CKD and monitor the decline of kidney function [4]. The majority of CKD patients are older than 60 years with early stage CKD and are mainly consulting in primary care, where the prevalence is estimated to be nearly 30% [5]. Only a few patients progress to ESRD (end stage renal disease) requiring dialysis [5, 6]. Referral to specialist care more than one to six months prior to initiation of dialysis was found to reduce mortality and hospitalisation, and improve preparation of dialysis [7].

Internationally, there are numerous clinical practice guidelines and recommendations on management of CKD, with varying referral criteria to specialist nephrology services, reflecting differences in health care systems [8]. The Kidney Disease Improving Global Outcomes (KDIGO, 2012), the British guideline from the National Institute of Clinical Excellence (NICE, 2014) guideline and the German College of General Practitioners and Family Physicians (DEGAM, 2019) recommend referral from stage 4 (GFR < 30 ml/min) onwards [4, 9, 10]. They also recommend referral with an GFR of 30–59 ml/min and varying additional criteria. The German Societies of Nephrology (DGfN) and Internal Medicine (DGIM) issued a short manual with recommendations in 2015 recommending referral with an eGFR < 45 ml/min [11]. In 2021 the NICE guideline was updated and suggested the use of kidney failure risk equation (KFRE) to estimate the 5-year risk of needing renal replacement therapy [12]. A risk of 5% is suggested as a threshold for referral.

The aim of referral criteria is to ensure timely and adequate access to nephrology services for patients with most at risk of complications or progression to ESRD and patients requiring specific treatment [13]. Avoiding unnecessary referral of low risk patients is usually not an explicitly stated goal, but is important, given the limited number of nephrologists, the high prevalence of CKD and the additional health care expenditures [14].

Specific referral recommendations often cannot be based on scientific evaluation. They rather reflect assumptions on acceptable referral thresholds or consensus of guideline authors and stakeholders from medical societies.

The aim of this study is to simulate and compare referral rate and corresponding health care expenditure of applying referral criteria according to the following guidelines and recommendations:

- Kidney Disease Improving Global Outcomes (KDIGO, 2012)
- National Institute for Health and Care Excellence (NICE, 2014)
- Recommendation of the German Society of Nephrology/German Society of Internal Medicine (DGfN/DGIM, 2015) and
- German College of General Practitioners and Family Physicians (DEGAM, 2019).

Additionally we calculated the ESRD risk for subjects with an eGFR < 60 ml/min using the KFRE as suggested by the update of the NICE guideline 2021, [12].

The second aim is to report the actually observed number of patients consulting a nephrologist.

## Methods

### Study design and population

**Fig. 1:**
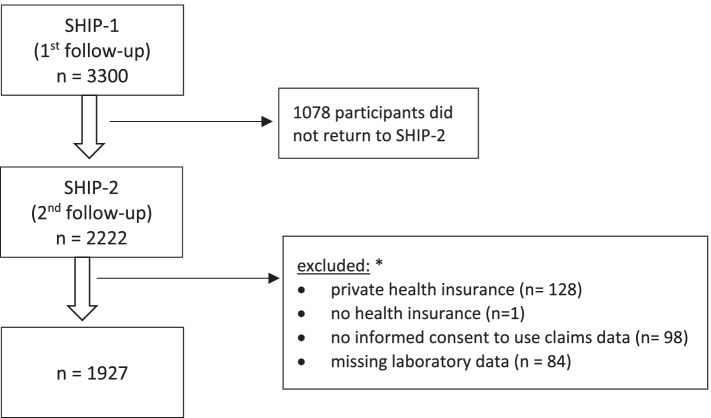
Flow chart of the study population selection. * some participants fulfilled multiple criteria

This is a simulation of applying different referral criteria suggested by various guidelines or recommendations to participants of a population-based cohort study in north-eastern Germany (Study of Health in Pomerania, SHIP). Participants were recruited with stratification for age and gender from random sample drawn from the local residence registry. This study provides clinical data of a population sample, independent of actual health service utilization. Data from participants attending the first as well as the second follow-up (*n* = 2222) were included (Fig. 1). Demographic data, somatometry data, standardised laboratory data, self-reported data from a computer assisted interview and data from the medication review were used. Data from the SHIP cohort were individually linked with claims data provided by the Association of Statutory Health Insurance Physicians in Mecklenburg-Vorpommern. Germany has universal health care coverage provided by statutory health insurance for 90% of the population. Ambulatory care in Germany, including specialist care is provided by private practices contracting with statutory health insurance [15]. Participants with private or no health insurance, participants who did not give informed consent to use claims data and participants with missing laboratory data were excluded from the analysis (Fig. 1). Claims data comprised ICD-10-GM diagnoses (German modification of the 10th revision of the International Classification of Diseases) and billing codes. Written informed consent was obtained from all subjects. The study protocol was approved by the Ethics Committee of the University Medicine Greifswald and can be examined elsewhere [16].

## Measurements and data analysis

We used laboratory data obtained within the frame of the SHIP study, not actual measurements obtained during medical care. Serum creatinine, age and sex (only race “white or other) were used to calculate estimated Glomerular filtration rate (eGFR) using the CKD-EPI-equation used during the observation period [17]. An eGFR category was assigned to each participant. Albuminuria categories were assigned using the calculated albumin-to-creatinine ratio (ACR). ICD-10-GM-coded diagnoses were used to define morphologic and structural kidney abnormalities (Table 1). Anatomical Therapeutic Chemical Classification System Codes (ATC Codes) were used to classify antihypertensive medication (C02-C09) using data from the medication review. For medication with combined components, the number of components were counted. Referral criteria were defined according to guideline recommendations using laboratory and blood pressure values (calculated as the mean of the 2^nd^ and 3^rd^ blood pressure measurement, systolic > 150 and/or diastolic > 90 mmHg), medication and billing codes (Table 1). Based on these criteria, we assigned a referral recommendation to each participant and estimated the proportion of participants eligible for referral according to each investigated guideline/recommendation. Additionally, billing codes were used to see who was actually referred and consulted a nephrologist within 1 year and 3 years prior to study examination (SHIP-2).

**Table 1:** Overview of criteria for referral recommendations

Guideline definition	Study data definition	Referral criteria			
		DEGAM	DGfN/DGIM	NICE 2014	KDIGO
Kidney function					
GFR	eGFR (ml/min)	< 30 **or** 30–59 ^b^ and one of the following criteria	< 45 **or** 45–59 and one of the following criteria	< 30 **or** 30–59 and one of the following criteria	< 30 **or** 30–59 and one of the following criteria
GFR progression > 5 ml/min / year	mean eGFR reduction > 5 ml/min from first to second follow-up examination		x		
GFR progression > 5 ml/min / year or GFR reduction ≥ 25% / year	mean eGFR reduction > 5 ml/min from first to second follow-up examination or mean eGFR reduction of ≥ 25%				x
GFR progression ≥ 15 ml/ min / year or GFR reduction ≥ 25% / year	mean eGFR reduction > 15 ml/min from first to second follow-up examination or mean eGFR reduction of ≥ 25%			x	
Hypertension refractory to treatment					
BP > 150 and/or > 90 mmHg and ≥ 3 antihypertensives	BP: mean systolic BP of 2. and 3. measurement Antihypertensives: ATC-codes C02-C09	x	x		
BP > 150 and/or > 90 mmHg and ≥ 4 antihypertensives	BP: mean systolic BP of 2. and 3. measurement Antihypertensives: ATC-codes C02-C09			x	x
Proteinuria					
Proteinuria > 200 mg/l, subjects with diabetes: > 20 mg/l	Urine albumin dipstick category, subjects with diabetes: urine protein dipstick category		x		
Albuminuria ≥ 300 mg/g or ≥ 30 mg/mmol	ACR				x
Proteinuria ≥ 70 mg/mmol and non-diabetic	ACR			x	
Albuminuria ≥ 30 mg/g or ≥ 3 mg/mmol	ACR	x			
ACR ≥ 30 mg/mmol and haematuria	ACR and urine dipstick category red blood cells + +			x	
Haematuria					
Micro- or macrohematuria	urine dipstick category red blood cells + + ^a^		x		x
Haematuria without known urologic cause	urine dipstick category red blood cells + + ^a^	x			
Morphologic and structural kidney abnormalities					
Morphologic kidney changes	ICD-10-GM codes • at least one billing code one year prior to study examination of second follow-up (N02.-, N20.-) • at least one billing code five years prior to study examination of second follow-up (N11.-, N13.-, N26.-, N28.-, C64.-, D41.0, Q61.-, Q63.-, I70.1)		x		
Kidney stones	ICD-10-GM codes N20.-, at least one billing code one year prior to study examination of second follow-up				x
Renal artery stenosis	ICD-10-GM code I70.1, at least one billing code five years prior to study examination of second follow-up			x	
Miscellaneous					
Hypocalcaemia	< 2.12 mmol/l		x		
Hyperphosphatemia	> 1.6 mmol/l		x		
Abnormalities of serum potassium	Serum potassium < 3.7 mmol/l or > 5.1 mmol/l				x
Inherited kidney disease	ICD-10-GM codes, at least one billing code five years prior to study examination of second follow-up (Q61.-, Q63.-)			x	x
Anaemia	WHO reference values haemoglobin, female: < 7.4 mmol/l, male: < 8.1 mmol/l		x		

*ACR* Albumin-Creatinin-Ratio, *ATC* Anatomical Therapeutic Chemical (ATC) Classification System Code, *BP* Blood Pressure, *eGFR* estimated Glomerular Filtration Rate, *ICD-10-GM* DEGAM: German society of general practice and family medicine, *DGIM* German society of internal medicine, *DGfN* German society of Nephrology, German modification of the 10th revision of the International Classification of Diseases, *NICE* National Institute of Health and Care Excellence, *KDIGO* Kidney Disease Improving Global Outcomes, *WHO* World Health Organisation

^a^ KDIGO criterium “erythrocyte cylinder or erythrocytes in spot urine > 20/high power field” was defined by substituting 1 µl for one high power field, which correlates to a 2 +  + urinary dip stick score in SHIP data

^b^ DEGAM made a good clinical practice point that younger patients with a low eGFR should be referred liberally, while in older patients (> 70 years old) with eGFR < 30 ml/min comorbidity, life expectancy and individual patient goals should be considered

In a second step, referral recommendations and age were used to estimate costs associated with nephrologist consultation. Costs were assigned based on billing codes and the official doctor’s fee scale of the National Association of Statutory Health Insurance Physicians from 2019 (billing codes 13,591 base fee schedule €26.62 for patients aged 6 to 59 years and 13,592 base fee schedule €27.60 for patients aged ≥ 60 years).

To estimate population level referral rates based on the SHIP cohort, the proportion of persons eligible for referral in the age categories 30 to 59 years and 60 to 90 years was multiplied with the number of persons in the same age categories according to the statistical office Mecklenburg-Vorpommern 2017 [18]. For pragmatic reasons, we assume that each patient is referred only once. Population level costs were estimated for each age category using costs as defined above. Descriptive analyses were performed using SAS Institute Inc., Cary, NC, USA, Software 9.4. This study has been reported in accordance with STROBE guidelines (Supplementary Table S2).

## Results

### Patient characteristics

A total of 1927 subjects (53% (1030/1927) female; median age = 59, Q_1_: 48; Q_3_: 69) were included in the analysis (Fig. 1). Of those, 2.8% (53/1927) had an eGFR < 45 ml/min (GFR category 3b, 4 or 5) and 7,1% (137/1927) an eGFR between 45–59 ml/min (GFR category 3a) (Table 2). Proteinuria was found in 2.1% (41/1927) of the entire study population. A blood pressure > 150 mmHg systolic and/or > 90 mmHg diastolic was measured in 24% (468/1927).

**Table 2:** Characteristics of the study population, *N* = 1927

Characteristic	**GFR category G1, G2 (60–89; ≥ 90 ml/min)**	**GFR category G3a (45–59 ml/min)**	**GFR category G3b (30–44 ml/min)**	**GFR category G4 (15–29 ml/min)**	**GFR category G5 (< 15 ml/min)**
	***n*** **= 1737 (90.1%)**	***n*= 137 (7.1%)**	***n*** **= 42 (2.2%)**	***n*** **= 9 (0.5%)**	***n*** **= 2 (0.1%)**
Female n (%)	930 (53.5)	76 (55.5)	20 (47.6)	3 (33.3)	1 (50)
Age, mean (± SD) (years)	56.7 (12.9)	73 (6.7)	77.5 (8.2)	80 (8.7)	65 (14.1)
Range (years)	31–89	56–93	57–90	66–89	55–75
Hypertension > 150 and/or > 90 mmHg, n (%)	401 (23.1)	49 (35.8)	14 (33.3)	3 (33.3)	1 (50)
Diabetes (self-reported), n (%)	194 (11.2)	37 (27)	16 (38.1)	6 (66.7)	1 (50)
Albuminuria ≥ 300 mg/g or ≥ 30 mg/mmol, n (%)	25 (1.4)	6 (4.4)	7 (16.7)	2 (22.2)	1 (50)
Anaemia, n (%)	104 (6)	21 (15.3)	15 (35.7)	3 (33.3)	1 (50)
Abnormalities of serum potassium, n (%)	84 (4.8)	15 (11)	9 (21.4)	1 (11.1)	1 (50)
Hypocalcaemia, n (%)	71 (4)	3 (2.2)	4 (9.5)	0 (0)	0 (0)
Morphologic kidney changes, n (%)	270 (15.5)	49 (35.7)	16 (38.1)	2 (22.2)	1 (50)
Kidney stones, n (%)	100 (5.8)	16 (11.7)	1 (2.4)	0 (0)	0 (0)
Inherited kidney disease, n (%)	120 (6.9)	24 (17.5)	6 (14.3)	0 (0)	0 (0)
Haematuria, n (%)	97 (5.6)	10 (7.3)	3 (7.1)	2 (22.2)	0 (0)

*GFR* Glomerular Filtration Rate, *SD* Standard deviation, morphologic kidney changes, kidney stones and haematuria are derived from claims data

Diabetes was self-reported by 13.2% (254/1927). Kidney stones were diagnosed in 6.1% (117/1927) and morphologic kidney changes in 17.5% (338/1927). The proportion of subjects with hypertension or diabetes was higher among subjects with reduced eGFR (Table 2).

### Participants eligible for referral

The overall proportion of participants eligible for referral to specialist nephrologist ranged from 4.9% according to DEGAM criteria to 8.3% according to the DGfN/DGIM criteria (Table 3). According to all guidelines/recommendations, the majority of patients eligible for referral were 60 years or older (> 95%, Table S1). In participants aged 60 years or older, DGfN/DGIM criteria yielded the highest proportion eligible for referral (16.5%) and DEGAM criteria the lowest (9.7%). Overall, estimated yearly cost based on a single referral to specialist nephrologist was between € 2,618 and € 4,384 (Table 3).

**Table 3:** Estimated referral rates according to different guidelines, the KFRE only and the corresponding costs, *N* = 1927

Guideline (year)	Participants eligible for referral n, (%)	Participants eligible for referral by age category, n (%)		estimated referral costs (€)^C^ according to referral criteria
		** < 60 years**^a^	** ≥ 60 years**^b^	
DGfN/DGIM (2015)	159/1927 (8.3)	4 (0.4)	155 (16.5)	4,384.48
KDIGO (2012)	148/1927 (7.7)	4 (0.4)	144 (15.3)	4, 080.88
NICE (2014)	103/1927 (5.4)	3 (0.3)	100 (10.6)	2,839.86
DEGAM (2019)	95/1927 (4.9)	4 (0.4)	91 (9.7)	2,618.08
KFRE (only)	15/1927 (0.8)	3 (0.3)	12 (1.3)	411.06
NICE 2021 (incl. KFRE)	104/1927 (5.4)	4 (0.4)	100 (10.6)	2,866.48

*DGfN/DGIM* German Society of Nephrology/German Society of Internal Medicine, *KDIGO* Kidney Disease Improving Global Outcomes, *NICE* National Institute for Health and Care Excellence, *DEGAM* German College of General Practitioners and Family Physicians

^a^ proportions based on *n* = 987 participants aged < 60 years, ^b^ proportions based on *n* = 940 participants aged ≥ 60 years. ^c^ Estimates based on a single specialist nephrologist consultation per person, excluding laboratory tests and ultrasound and biopsy, €26.62/consultation for patients < 60 years, €27.60/consultation for patients ≥ 60 years

### Patients actually consulting a nephrologist

**Fig. 2:**
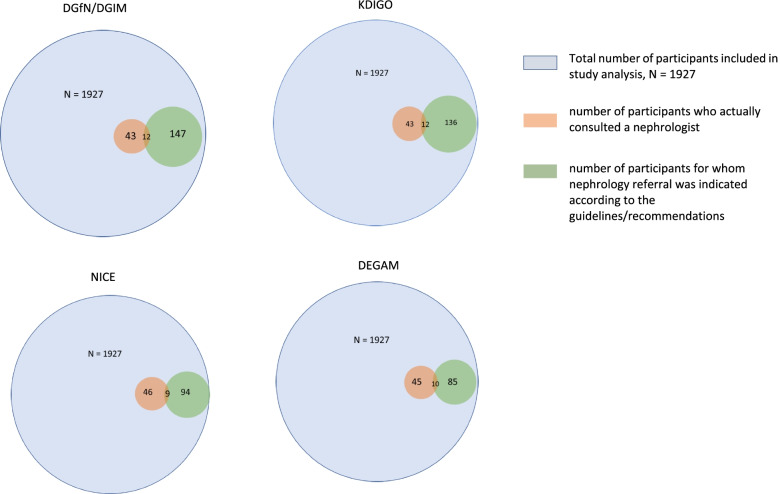
Diagram showing participants referred to a nephrologist according to guidelines/recommendations and participants actually consulting a nephrologist within 1 year prior to study examination (SHIP-2). DGfN/DGIM: German Society of Nephrology/German Society of Internal Medicine; KDIGO: Kidney Disease Improving Global Outcomes; NICE: National Institute for Health and Care Excellence; DEGAM: German College of General Practitioners and Family Physicians

A total of 55/1927 (2.9%) subjects actually consulted a nephrologist (GOP 13,591, GOP 13,592) within 1 year prior to study examination (SHIP-2). Of those, 33 participants consulted only once, 13 participants twice, 6 participants 3 times and 3 participants 4 times within 1 year. Within 3 years prior to study examination, 75/1927 (3.9%) subjects consulted a nephrologist. Of those consulting a nephrologist within 1 year prior to study examination (*n* = 55), 12 patients fulfilled referral criteria according to DGfN/DGIM and KDIGO, 9 patients according to NICE criteria and 10 patients according to DEGAM criteria (Fig. 2). Participants eligible for referral according to the DGfN/DGIM criteria were older (median age 75 years) and had a lower eGFR (median 50 ml/min) than those who were actually referred and consulted a nephrologist (median age 57 years, median eGFR 91 ml/min). Of the 190 participants with an eGFR < 60 ml/min a total of 15 had a risk > 5% for ESRD according to KFRE, but only 3, mostly younger patients (< 60 years old) had a nephrology consultation. The average age of the non-referred patients was 78 years (range 58–90).

### Estimated population level referral and corresponding costs for Mecklenburg-Vorpommern

There were 675,871 persons aged 30–59 and 508,177 persons aged 60–90 living in the state Mecklenburg-Vorpommern in the year 2017 [18]. Based on the referral rates according to different guidelines (Table 3), estimated population level total cost for nephrologist referral amounted to € 1,432,440 (DEGAM) and reached up to € 2,386,186 (KDIGO) (Table 4).

**Table 4:** Estimated population level referral rates for Mecklenburg-Vorpommern, Germany and the corresponding cost

Guideline (year)	Eligible for referral by age category n (%)		Referral cost (€^a^)		Total cost (€^a^)
	**Aged < 60**	**Aged ≥ 60**	**Aged < 60**	**Aged ≥ 60**	
DGfN/DGIM (2015)	2,703 (0.4)	83,849 (16.5)	71,953.86	2,314,232.40	2,386,186.26
KDIGO (2012)	2,703 (0.4)	77,751 (15.3)	71,953.86	2,145,927.60	2,217,881.46
NICE (2014)	2,027 (0.3)	53,866 (10.6)	53,958.74	1,486,701.60	1,540,660.34
DEGAM (2019)	2,703 (0.4)	49,293 (9.7)	71,953.86	1,360,486.80	1,432,440.66

^a^Estimates based on 675,871 people < 60 years old; 508,177 ≥ 60 years old, and a single specialist nephrologist consultation per person, excluding laboratory tests and imaging; €26.62/consultation for patients < 60 years, €27.60/consultation for patients ≥ 60 years

*DGfN/DGIM* German Society of Nephrology/German Society of Internal Medicine, *KDIGO* Kidney Disease Improving Global Outcomes*, NICE* National Institute for Health and Care Excellence, *DEGAM* German College of General Practitioners and Family Physicians

## Discussion

### Summary of the main results

The aim of our analysis was to estimate and compare actual consultation rate and recommended referral rates to specialist nephrology services as well as corresponding costs, based on different guideline recommendations. The proportion of subjects meeting referral criteria and associated cost differed between guidelines. DEGAM criteria yielded an overall referral rate of 4.9%, while applying DGfN/DGIM criteria resulted in a referral rate of 8.3%. In subjects older than 60 years, differences were even more pronounced, and rates ranged from 9.7% (DEGAM) to 16.5% (DGfN/DGIM). The observed actual nephrology consultation rate (2.9%) was lower and there was mismatch between eligible and referred subjects (Fig. 2). The estimated population level total costs for implementing referral criteria in the state Mecklenburg-Vorpommern, Germany, varied between € 1,432,440 (DEGAM) and € 2,386,186 (DGfN/DGIM) based upon a population of almost 1.2 million, but only considering a single specialist nephrology consultation.

### Meaning of the results and comparison with literature

CKD is one of the fastest growing chronic diseases due to demographic changes [1, 3]. Moreover, the prevalence of obesity, diabetes and hypertension is increasing worldwide, which will potentially increase CKD prevalence even further [1]. Since age related decline in GFR is assumed to be an important driver of the increase in prevalence, a call for an age-adapted definition of CKD has been made [19]. This aspect has been taken into consideration by the DEGAM guideline which made a good clinical practice point for individual assessment of the benefits of nephrology referrals in older patients with concurrent morbidity. However, the available data did not allow to make such judgements. We assume we overestimate the number of patients for whom a referral would be of clinical benefit. This might also explain the lower proportion of observed actual nephrology consultations.

Measurements of albumin-to-creatinine ratio (ACR) are not frequently performed in ambulatory are [20]. In our sample we identified subjects with proteinuria according to ACR measurement within the frame of the cohort study. We can therefore assume that the degree or proteinuria was unknown to the treating physicians. This explains on the one hand the higher proportion of referrals according to recommendations and on the other hand the mismatch between consulting and referred patients.

In Germany, the prevalence of CKD in the adult population under 80 years is estimated to range between 2.3% based on GFR and 11.5% based on proteinuria, resulting in more than 10 million of the general population and more than 50% of nursing home residents meeting the criteria for CKD [6, 21]. Due to the high prevalence of CKD in combination with ultimately limited resources in the healthcare system in terms of specialist care services and cost, referral criteria for CKD have important implications for health care resource allocation [14].

Late referral to specialist nephrology services has been reported and associated with worse outcomes [22]. It has to be considered that emergency dialysis or progression to ESRD is not always preventable. This is on the one hand due to no prior medical contact, rapid and unpredictable decline in renal function or acute health conditions [23, 24]. On the other hand, progression to ESRD occurs in some patients despite medical care. It is assumed, that early referral defined as 6 or 12 months prior to renal replacement therapy or referral at GFR < 60 ml/min (CKD stage 3a) could potentially contribute to delay progression and improve prognosis in patients with CKD [7, 25]. These assumptions were based on retrospective data of patients with end stage renal disease and dialysis, where early referral was found to be associated with better preparation and placement of dialysis access, while improved care was defined according to frequency of blood pressure measurements and management of diabetes [25]. Although these are important factors in CKD management, retrospective analyses of this kind based on the small fraction of CKD patients who will reach ESRD, cannot be used to draw conclusions about the wider population of predominantly older patients with stable CKD [26]. In fact, the challenge is to distinguish between the large number of CKD patients with stable or slowly declining kidney function and low lifetime ESRD risk and patients who require specialist care because of likely progression to ESRD or treatment of uremic complications such as CKD-MBD, renal anaemia or metabolic acidosis.

Since the majority of studies regarding CKD have been conducted in clinical settings and with high risk patients, formulating referral criteria for primary care and other low risk settings is challenging [13]. Recent guidelines have tried to formulate referral criteria aimed at distinguishing between patients with low versus high risk for ESRD [4, 8–11]. Analyses of estimated implications of KDIGO referral criteria for the US indicate, that referral criteria do not effectively distinguish between high and low risk patients, when applied to primary care or population based cohorts [27]. It was estimated, that implementing KDIGO referral criteria in a primary care population would result in a 38% increase in total nephrology patient volume and a 67% increase in new referrals, leading to a supply–demand mismatch of available workforce and resources [27, 28]. This is in line with the results of our analysis, where implementing KDIGO criteria would lead to more than double increase in referral rate compared to actual referral (7.7% vs. 3%).

KDIGO, DGfN/DGIM and NICE (2014) criteria aim to identify high risk subgroups by proposing additional criteria for CKD patients with reduced GFR, including haematuria, albuminuria and refractory hypertension. The differences between the referral rates of the different criteria are small. We assume that criteria do not succeed in distinguishing high-risk subgroups in older patients, resulting in excessive referral rates of 16.5% (DGfN/DGIM) and 10.6% (NICE 2014) in persons aged ≥ 60 years. Implementing DEGAM criteria, resulted in the lowest overall referral rate of 5% at the population level, but even the strict application of these criteria resulted in a 9.7% referral rate in patients aged ≥ 60 years. This has implications if referral criteria are used for quality measurements. In our analysis, only a minority of participants referred, fulfilled the stipulated referral criteria (Fig. 2). The participants actually referred had a higher eGFR and were younger than those who were not referred but were eligible for referral. Our results show that the DGfN/DGIM criteria are not used in clinical practice.

Although our data set exceeds data usually available, in which ACR is often not included [20], the data do not allow to fully assess the appropriateness of selection of patients who are most likely to benefit from specific nephrology services. The KFRE identified the lowest proportion of participants eligible for nephrology referral, but only 3 out of 15 (20%) actually received a referral. This should be interpreted cautiously since other relevant comorbidities or individual arrangements cannot be excluded. It is conceivable that the risk of ESRD was underestimated in some participants, where treating physicians were not aware of the amount of proteinuria. One should keep in mind that NICE 2021 suggests additionally other criteria like decrease eGFR > 25% in 12 months, suspected genetic disease and suspected renal artery stenosis [12].

Ambulatory physicians should measure ACR more frequently to make referral decisions. This has implications for monitoring quality of medical services based on current referral recommendations. If risk estimation with the KFRE, as proposed by the updated NICE guideline, increased measurements of ACR in primary care can be expected.

There are limited specific treatment options requiring specialist care for most patients, improving CKD prognosis [29, 30], apart from hypertension and diabetes management, which are recommended regardless of kidney function, and despite recent progress with Sodium-Glucose Cotransporter-2 (SGLT2) Inhibitors. Therefore, there is no rationale for mandatory early referral of patients with uncomplicated or early CKD stages or with stable GFR considering patients age. Management of these patients can be provided by general practitioners and clinical practice guidelines on blood pressure and diabetes management are available. This approach would yield a potential cost reduction while preserving quality of care and should be complemented by conservative referral criteria, accounting for age, comorbidity and risk of ESRD. At the same time, general practitioners describe a need for informal shortcuts for specialist advice when faced with older, multimorbid patients with conflicting health care needs and low lifetime risk of ESRD [24, 31].

### Recommendations for research and future guideline development

Due to the strong association of kidney function with age (age dependent decline), there is a need of an age-dependent approach to management and referral, incorporating CKD prognosis and comorbidities. Proposed age-adjusted CKD criteria were postulated by Delanaye et al. (2019), based on a meta-analysis of mortality risk in different eGFR stages [19]. Applying these criteria might be an additional factor in identifying patients with high ESRD risk from those with age related kidney function decline. Prospective population based analyses on the natural history of CKD, mortality and clinically relevant endpoints in a low-risk or primary care setting are scarce [32]. Recently, the Kidney Failure Risk Equation (KFRE) for predicting the 2 and 5 year probability of ESRD was successfully validated in a British primary care setting and a large Canadian study population [32–34]. Based on this equation, tools to recommend referral to a nephrologist were developed and could be used to optimise referral recommendations [35]. This has already led to a change in the updated NICE Guideline 2021 which recommends the use of the KFRE instead of an eGFR on its own for referral recommendation [12]. Further research, ideally based on large, prospective, primary care-based cohorts, would be needed to further validate evidence-based referral criteria and existing prediction tools. Future guidelines should emphasize risk for ESRD and life expectancy in referral recommendations rather than fixed eGFR values [30]. Research has shown, that treatment burden is significant in patients with CKD. Future research should address the role of specialist referral on burden of disease and quality of life in CKD patients in the German healthcare setting [36, 37].

### Strengths and limitations

This is the first study to our knowledge simulating the implications of applying different referral criteria for CKD in a German population. Our analysis is based on population-based data, which allowed to consider ACR, which is not routinely measured. However, it is limited due to the attrition bias (loss-to-follow up). We might underestimate the number of referred patients, since we cannot exclude that some patients received a referral but did not actually consult. We assume that true costs are much higher than calculated, because billing codes did not reflect all cost associated with referral (laboratory tests, ultrasound) and in reality, multiple follow up visits are common. Since this limitation affects all calculated guideline referral rates, our conclusions from the comparison between guidelines are not affected. We conducted a complete case analysis. Billing data was only available for subjects with statutory health insurance and for subjects who gave consent to use their claims data (Fig. 1). Nevertheless, study results are based on 1927 study participants. GFR progression was not available on a yearly basis. Therefore, we used the 5-year progression of > 5 ml/min or ≥ 15 ml/ min or ≥ 25% corresponding to the guideline criteria. Our simulation does not allow to assess over- or underutilization of nephrology services or harm to the participants due to the different referral criteria. We have no long-time follow up data regarding renal outcome.

## Conclusions

Applying different proposed referral criteria for CKD patients to specialist nephrology services results in differences in referral rates and costs. Referral rates exceed actually observed consultation rates. Referral criteria need to be evaluated in terms of available workforce and resources but also regarding over- and underutilization of nephrology services.

## Supplementary Information

**Additional file 1:** **Table S1** Estimated referral rates according to different guidelines, age proportion calculated as row percentage. **Table S2** STROBE Statement – Checklist of items that should be included in reports of cohort studies.

## Data Availability

Billing Data cannot be shared publicly due to legal restrictions regarding claims data according to SGB XI. SHIP data are available on reasonable request according the bylaws of the research association of the community medicine https://www.fvcm.med.uni-greifswald.de/dd_service/data_use_intro.php.
