# Supplementary material for: Referral criteria for chronic kidney disease: implications for disease management and healthcare expenditure—analysis of a population-based sample
Source: BMC Nephrol. 2022 Jun 24;23:225. doi: 10.1186/s12882-022-02845-0 (PMC9229756; doi:10.1186/s12882-022-02845-0)
Supplement: Supplementary file 1 — Additional file 1: Table S1 Estimated referral rates according to different guidelines, age proportion calculated as row percentage. Table S2 STROBE Statement – Checklist of items that should be included in reports of cohort studies. [file 12882_2022_2845_MOESM1_ESM.docx]

Supplements

Table S1 Estimated referral rates according to different guidelines, age proportion calculated as row percentage

| Guideline | Participants eligible for referral, n (%) | Participants eligible for referral by age category^1^, n (%) | | |
| --- | --- | --- | --- | --- |
|  |  | **< 60 years** |  | **≥ 60 years** |
| DGfN/DGIM (2015) | 159 (8.3) | 4 (2.5) |  | 155 (97.5) |
| KDIGO (2012) | 148 (7.7) | 4 (2.7) |  | 144 (97.3) |
| NICE (2014) | 103 (5.4) | 3 (2.9) |  | 100 (97.1) |
| DEGAM (2019) | 95 (4.9) | 4 (4.2) |  | 91 (95.8) |

^1^Proportions calculated as row percentages

^2^Estimates based on a single specialist nephrologist consultation per person, excluding laboratory tests and imaging

DGfN/DGIM: German Society of Nephrology/German Society of Internal Medicine; KDIGO: Kidney Disease Improving Global Outcomes; NICE: National Institute for Health and Care Excellence; DEGAM: German College of General Practitioners and Family Physicians

**Table S2** STROBE Statement – Checklist of items that should be included in reports of cohort studies

|  | Item No | Recommendation | page number |
| --- | --- | --- | --- |
| **Title and abstract** | 1 | (*a*) Indicate the study’s design with a commonly used term in the title or the abstract | 2 |
|  |  | (*b*) Provide in the abstract an informative and balanced summary of what was done and what was found | 2 |
| Introduction | | |  |
| Background/rationale | 2 | Explain the scientific background and rationale for the investigation being reported | 3-4 |
| Objectives | 3 | State specific objectives, including any prespecified hypotheses | 4 |
| Methods | | |  |
| Study design | 4 | Present key elements of study design early in the paper | 4 |
| Setting | 5 | Describe the setting, locations, and relevant dates, including periods of recruitment, exposure, follow-up, and data collection | 4-5 |
| Participants | 6 | (*a*) Give the eligibility criteria, and the sources and methods of selection of participants. Describe methods of follow-up | 4-5 |
|  |  | (*b*) For matched studies, give matching criteria and number of exposed and unexposed |  |
| Variables | 7 | Clearly define all outcomes, exposures, predictors, potential confounders, and effect modifiers. Give diagnostic criteria, if applicable | 5-6 |
| Data sources/ measurement | 8* | For each variable of interest, give sources of data and details of methods of assessment (measurement). Describe comparability of assessment methods if there is more than one group | *4-5* |
| Bias | 9 | Describe any efforts to address potential sources of bias | 6 |
| Study size | 10 | Explain how the study size was arrived at | Figure 1 |
| Quantitative variables | 11 | Explain how quantitative variables were handled in the analyses. If applicable, describe which groupings were chosen and why | 5 |
| Statistical methods | 12 | (*a*) Describe all statistical methods, including those used to control for confounding | 5-6 |
|  |  | (*b*) Describe any methods used to examine subgroups and interactions | - |
|  |  | (*c*) Explain how missing data were addressed | 5, Fig. 1 |
|  |  | (*d*) If applicable, explain how loss to follow-up was addressed | 5, Fig. 1 |
|  |  | (*e*) Describe any sensitivity analyses | - |
| Results | | |  |
| Participants | 13* | (a) Report numbers of individuals at each stage of study—eg numbers potentially eligible, examined for eligibility, confirmed eligible, included in the study, completing follow-up, and analysed | Fig. 1 |
|  |  | (b) Give reasons for non-participation at each stage | Fig. 1 |
|  |  | (c) Consider use of a flow diagram | Fig. 1 |
| Descriptive data | 14* | (a) Give characteristics of study participants (eg demographic, clinical, social) and information on exposures and potential confounders | Table 2, page 7 |
|  |  | (b) Indicate number of participants with missing data for each variable of interest | - |
|  |  | (c) Summarise follow-up time (eg, average and total amount) | - |
| Outcome data | 15* | Report numbers of outcome events or summary measures over time | 7-8 |
| Main results | 16 | (*a*) Give unadjusted estimates and, if applicable, confounder-adjusted estimates and their precision (eg, 95% confidence interval). Make clear which confounders were adjusted for and why they were included | - |
|  |  | (*b*) Report category boundaries when continuous variables were categorized | - |
|  |  | (*c*) If relevant, consider translating estimates of relative risk into absolute risk for a meaningful time period | - |
| Other analyses | 17 | Report other analyses done—eg analyses of subgroups and interactions, and sensitivity analyses | - |
| Discussion | | |  |
| Key results | 18 | Summarise key results with reference to study objectives | 8 |
| Limitations | 19 | Discuss limitations of the study, taking into account sources of potential bias or imprecision. Discuss both direction and magnitude of any potential bias | 13 |
| Interpretation | 20 | Give a cautious overall interpretation of results considering objectives, limitations, multiplicity of analyses, results from similar studies, and other relevant evidence | 9-12 |
| Generalisability | 21 | Discuss the generalisability (external validity) of the study results | - |
| Other information | | |  |
| Funding | 22 | Give the source of funding and the role of the funders for the present study and, if applicable, for the original study on which the present article is based | 16 |
